# A cluster randomised controlled trial to investigate the effectiveness and cost effectiveness of the ‘Girls Active’ intervention: a study protocol

**DOI:** 10.1186/s12889-015-1886-z

**Published:** 2015-06-04

**Authors:** C L Edwardson, D M Harrington, T Yates, D H Bodicoat, K Khunti, T Gorely, L B Sherar, R T Edwards, C Wright, K Harrington, M J Davies

**Affiliations:** Diabetes Research Centre, University of Leicester, Leicester, UK; NIHR Leicester-Loughborough Diet, Lifestyle, and Physical Activity Biomedical Research Unit, Leicester, UK; School of Sport, University of Stirling, Scotland, UK; School of Sport, Exercise and Health Sciences, Loughborough University, Leicestershire, UK; Centre for Health Economics and Medicines Evaluation, Bangor University, Bangor, UK; Youth Sport Trust, Leicestershire, UK

**Keywords:** Physical activity, Peers, Accelerometer, Adolescent, Girls, Intervention, Cost-effectiveness

## Abstract

**Background:**

Despite the health benefits of physical activity, data from the UK suggest that a large proportion of adolescents do not meet the recommended levels of moderate-to-vigorous physical activity (MVPA). This is particularly evident in girls, who are less active than boys across all ages and may display a faster rate of decline in physical activity throughout adolescence. The ‘Girls Active’ intervention has been designed by the Youth Sport Trust to target the lower participation rates observed in adolescent girls. ‘Girls Active’ uses peer leadership and marketing to empower girls to influence decision making in their school, develop as role models and promote physical activity to other girls. Schools are provided with training and resources to review their physical activity, sport and PE provision, culture and practices to ensure they are relevant and attractive to adolescent girls.

**Methods/Design:**

This study is a two-arm cluster randomised controlled trial (RCT) aiming to recruit 20 secondary schools. Clusters will be randomised at the school level (stratified by school size and proportion of Black and Minority Ethnic (BME) pupils) to receive either the ‘Girls Active’ intervention or carry on with usual practice (1:1). The 20 secondary schools will be recruited from state secondary schools within the Midlands area. We aim to recruit 80 girls aged 11–14 years in each school. Data will be collected at three time points; baseline and seven and 14 months after baseline. Our primary aim is to investigate whether ‘Girls Active’ leads to higher objectively measured (GENEActiv) moderate-to-vigorous physical activity in adolescent girls at 14 months after baseline assessment compared to the control group. Secondary outcomes include other objectively measured physical activity variables, adiposity, physical activity-related psychological factors and the cost-effectiveness of the ‘Girls Active’ intervention. A thorough process evaluation will be conducted during the course of the intervention delivery.

**Discussion:**

The findings of this study will provide valuable information on whether this type of school-based approach to increasing physical activity in adolescent girls is both effective and cost-effective in the UK.

**Trial registration:**

ISRCTN10688342. Registered 12 January 2015.

## Background

Physical activity has been associated with numerous physiological and psychological benefits for young people [1, 2]. Despite this, a large proportion of children and adolescents do not meet UK recommended physical activity guidelines of at least 60 min of moderate to vigorous physical activity (MVPA) every day [3]. Self-reported data from the Health Survey for England reports that only 18 % of young people aged 5–15 years meet the recommended guidelines. Furthermore, declines in MVPA are evident during and following the transition to secondary school (age 11+ years) when a significant drop in the number of both boys and girls meeting MVPA guidelines is observed, with the drop often being steeper in girls. For example, at age 5 to 7 the same proportion of boys and girls meet recommendations (~24 %) but this declines to 14 % in boys and 8 % in girls by age 13 to 15 [4].

UK adolescent girls cite a number of personal factors (body image, embarrassment, skill), perceptions about physical activity (too competitive, not fun) and a perceived lack of support (limited opportunities and provision designed for girls, boys getting more support) as barriers to being active [5]. These issues tend to be exacerbated as girls move through adolescence [5], a time when dramatic changes in physical maturation has psychological and social impact that could contribute to girls’ disengagement from physical activity [6]. Due to the pronounced gender differences in physical activity levels, developing physical activity interventions and strategies that are sensitive to girls’ needs and interests is a challenging priority [7].

To date, a variety of settings and methods have been used to encourage physical activity in young people, including alterations to the school cultural or physical environment, modifying the school curriculum, adding extra physical activity within the school day, and interventions with a community or family component [8–11]. Reviews to date have struggled to draw general conclusions on the effectiveness of physical activity interventions among girls due to inconsistent findings, but several strategies have consistently emerged as being associated with enhanced efficacy. For example, interventions appeared to be most effective when they were school-based, included enjoyable physical education (PE) as a main component (i.e., making PE more enjoyable for girls by increasing choice and non-competitive and innovative activities) and promoted positive peer relationships through peer tutoring or peer modelling and social support of friendships groups in physical activity settings [8, 11]. Other research has also suggested that as children move into adolescence, peers become a strong influence on physical activity [12, 13]. One review identified only two peer based interventions aimed at young girls; suggesting that future research is needed to evaluate these types of programmes especially when implemented with older girls [8]. Reviews identified limitations in the methodological quality of the evaluation such as lack of high quality randomised controlled trials, lack of precision of the physical activity outcome measures and small sample sizes [8, 11]. Furthermore, the majority of school-based evidence comes from North America and elsewhere [9, 10, 14–20] and differences in infrastructure, school systems and culture make it inappropriate to translate these directly to the UK [8, 11, 21]. There is a small evidence base for school based interventions in the UK but this is limited and focuses mainly on primary schools [22–27], or increasing activity solely during PE classes [28, 29].

This study seeks to build on previous successful school-based interventions by robustly evaluating through a randomised controlled trial a school-based intervention (Girls Active) which focuses on increasing adolescent girls’ physical activity, sport and PE participation through peer leadership and marketing and engaging girls in decision making within the school for physical activity, sport and PE provision. A wider whole school approach through training for teachers, teachers reviewing current physical activity, PE and sport provision for girls, setting action plans and on-going mentoring for schools is also incorporated.

### Aim and objectives of the study

#### Aim

To investigate whether ‘Girls Active’ is effective and cost-effective in promoting increased physical activity in adolescent girls.

### Primary objective

1. To investigate whether ‘Girls Active’ leads to higher objectively measured moderate-to-vigorous physical activity (MVPA) in adolescent girls aged 11–14 years at 14 months after baseline assessment compared to the control group.

### Secondary objectives

2. To investigate whether ‘Girls Active’ results in changes to the following outcomes at 7 and 14 months after baseline assessment:
  Increases in objectively measured total volume of physical activity.
  An increase in the proportion of girls meeting MVPA guidelines (measured objectively).
  Increases in objectively measured MVPA.
  Reductions in time spent sedentary (measured objectively and self-reported).
  Reductions in measures of adiposity (body mass index percentile, percent body fat).
  Improvements in psychological factors that may mediate physical activity participation (attitudes to physical activity, enjoyment of physical activity, motivation and confidence to be active, activity-related social support, physical self-perceptions).

3. To determine the cost-effectiveness of the ‘Girls Active’ intervention.

## Methods

### Design

**Fig. 1:**
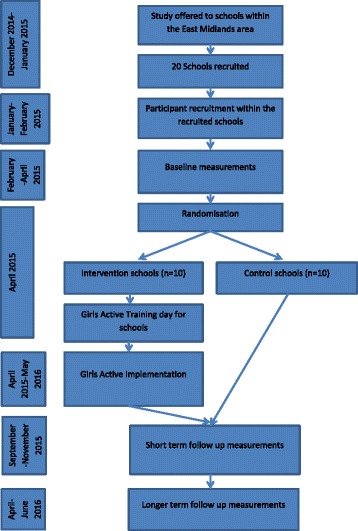
Flow diagram of study

This study is a two-arm cluster randomised controlled trial (RCT) aiming to recruit 20 secondary schools. Clusters will be randomised at the school level. The 20 secondary schools will be recruited from state secondary schools within the Midlands area (i.e., Leicestershire, Nottinghamshire, Derbyshire and Warwickshire). We aim to recruit 80 girls aged 11–14 years in each school. Figure 1 shows the flow of schools and participants through the study.

This study will be conducted, analysed and reported according to the Consolidation Standards of Reporting Trials (CONSORT) statement for cluster RCTs [30]. Ethical approval has been sought and obtained from the University of Leicester ethical representative and the University of Leicester will act as study sponsor.

### School recruitment

All state secondary schools in Leicester, Leicestershire and Rutland with girls aged 11–14 years (*n* = 56) will be invited to take part in the study along with a small number of other state secondary schools along the M1 corridor in Derbyshire, Nottinghamshire and Warwickshire. Eligible schools will be sent an initial letter outlining the ‘Girls Active’ intervention and the evaluation and inviting them to a briefing event. At the briefing event the teachers will receive a detailed presentation about the ‘Girls Active’ intervention, the study evaluation methods and the requirements of being involved. At the end of briefing event the school representative will be given written information for the head teacher along with a consent form. If schools are interested in being involved they will return the consent form signed by the head teacher.

All schools involved in the study will receive a £500 honorarium at the end of the study (i.e., once they have completed all evaluation measures) to encourage participation in the study and discourage dropout.

### Participant recruitment

Following school recruitment, all eligible participants within each school will be invited to take part in the study. All girls aged 11–14 years are eligible to take part and will be provided with an invitation pack, containing an invitation letter to parent(s)/guardian(s), parent/guardian information sheet, an opt out consent form for the parent/guardian (the parent/guardian only returns a signed opt out consent form if they do not want their child to participant) and a participant information sheet for the girls. If more than 80 girls are able to take part (i.e., have not returned an opt out consent form from their parent/guardian), 80 girls will be randomly selected using a computer generated number system. These 80 girls will provide verbal assent for study participation (at baseline and at each follow up visit).

### Sample size

Sample size calculations were performed to detect a mean difference of ten minutes of MVPA between the intervention and control groups. In order to detect a difference of 10 min/day between groups, assuming a standard deviation of 18 min in MVPA [31], a power of 90 %, a significance of 0.05, a cluster size of 56 girls and an intra-class correlation of 0.1, the sample size needed is 18 schools, increasing to 20 schools (10 schools per group) to allow for cluster attrition. To allow for 30 % loss to follow up and non-compliance with the accelerometer we will recruit 80 girls per cluster (1600 girls in total).

### Randomisation

Randomisation will occur after baseline assessments and will be carried out by the Leicester Clinical Trials Unit. Randomisation will occur at the school level (stratified by school size and proportion of Black and Minority Ethnic (BME) pupils) to receive either the ‘Girls Active’ intervention or carry on with usual practice (1:1).

### Intervention

The Youth Sport Trust (YST), an independent charity, have spent the last 10 years developing and refining initiatives aimed at engaging young girls in physical activity, physical education (PE) and sport. This expanse of work has evolved into a programme called ‘Girls Active’. ‘Girls Active’ is focused on providing a support framework to schools to review their physical activity, sport and PE provision, culture and practices to ensure they are relevant and attractive to all adolescent girls but with a particular focus on 11–14 year old girls (Key Stage 3). Furthermore, ‘Girls Active’ uses peer leadership and marketing to empower girls to influence decision making in their school, develop as role models and ‘sell’ physical activity to other girls. This process is underpinned by teachers and girls working together to understand the preferences and motivations of girls to take part in physical activity, sport and PE. ‘Girls Active’ is designed to be a flexible process for delivery but there are several key elements that underpin the programme. The elements are listed below.

1. **Self-evaluation and mission analysis -** ‘Girls Active’ draws on the highly effective Mission 2012 review, planning and evaluation framework used by UK Sport to generate sporting success at the London Olympic and Paralympic Games. Using an adaptation of this Mission Analysis framework and a combination of marketing principles and youth leadership, it helps schools to review their existing culture and practice and to deliver an action plan tailored to their girls’ needs. This is an exercise that is carried out as a ‘pre-intervention and training’ task and allows schools to reflect on their practice that currently exists within their school.
2. **Training for school leads –** Schools are invited to a one day orientation and training day to introduce schools leads to the resources and action planning This training covers the impact on school development plans and how using interventions such as ‘Girls Active’ can have a successful impact on attainment and achievement and on certain student groups that schools are trying to engage. The teachers also share challenges, successes and ideas with each other.
3. **Package of resources –** At the training day schools receive a package of resources from the YST aimed at the teachers and the peer leadership and marketing group. This package contains resources such as marketing plans, an action planning guide, case studies, still and video images to stimulate discussion, and a ‘Making it Yours’ branding toolkit for peer leaders including a CD with logos, graphics and designs that peer leaders and teachers can use in their campaign.
4. **Peer leadership and marketing group –** School leads can encourage, invite or ask for expressions of interest from Key Stage 3 girls (11-14 years) to volunteer to be part of this group. They are commonly girls who are not necessarily engaged in sporting and physical activities or particularly enthusiastic about participation, but are often girls who would be seen as leaders for non-sporting reasons and thus could have a positive influence on their peers. This group will influence decision making in their school, develop as role models, promote physical activity to other girls and run peer led physical activity sessions and events. Those involved in the peer leadership and marketing group will be provided with the branding toolkit and marketing ideas and will develop the campaign with support from the school lead.
5. **Using the student ‘voice’ to develop and market ideas for change –** The ‘voice’ of the adolescent girl is key in decisions about physical activity, PE and sport in the school including the provision of changing facilities, kit, activity content, programming, inclusion and imagery. This process is underpinned by teachers and girls working together to come up with innovative and alternative physical activity and sports that they would like to participate in and can be incorporated in PE and extra-curricular activities. Most importantly this is a ‘different type’ of student voice, one that probably hasn’t been used before as it calls on the base of students who would not traditionally be involved in this type of provision.
6. **On-going support and mentorship from the Health and Wellbeing School and the YST –** The local Health and Wellbeing School in Leicester, UK will offer support and mentorship to the intervention schools. This on-going support throughout the intervention phase could involve phone or email support and one to one visit support as is required. Their experience of developing and implementing similar programmes in schools that have little experience will be crucial to the on-going success of the ‘Girls Active’ programme. This mentor hub will also work alongside the programme manager responsible for ‘Girls Active’ within the YST.
7. **Peer review day -** All schools will be invited to a peer review day to identify learning and practice; this will be led by the YST and the Health and Wellbeing School with the aim of teachers and peer leadership and marketing groups coming together to share ideas and solutions. This will tie in with the schools’ ‘mission analysis’ self-review and allow schools to progress those areas of development that have been highlighted through their involvement in ‘Girls Active’.
8. **Funding for capacity building within the school -** There will be capacity payments made available to the schools involved in the ‘Girls Active’ intervention. Each of the intervention schools will be given capacity funding of £1000 in two £500 instalments.

The ‘Girls Active’ programme is guided by social cognitive theory (SCT) [32]. The literature on physical activity in young people suggests that addressing multiple levels of influence on behaviour (i.e. from the individual level to the environmental level), creating choice, increasing access and availability and physical opportunities to be active and fostering social support through positive peer relationships or friends are important and key during adolescence [8]. These constructs are all embedded within SCT and explicitly incorporated into the ‘Girls Active’ programme. Other core constructs of SCT such as observational learning, self-regulation and self-monitoring are also built into key intervention activities.

### Measurements

Data will be collected at three time points; baseline (February-April 2015), seven months after baseline (September-November 2015) and 14 months after baseline (April-June 2016). Trained researchers, following standard operating procedures, from University of Leicester and Loughborough University will collect data within schools and will be blind to the control and intervention allocation.

### Objectively measured physical activity

Participants will wear the GENEActiv Original accelerometer (Activinsights Ltd, Kimbolton, UK) continuously (i.e., 24 h/day) for seven days on their non-dominant wrist. This device has been found to be a valid and reliable objective measure of physical activity in young people [33]. Participants will be provided with a £5 gift voucher on return of their accelerometer with at least four valid days of data. The following variables will be calculated from the accelerometer data:

1. Mean minutes of MVPA (overall and weekday and weekend split)
2. Mean total volume of activity per day (overall and weekday and weekend split)
3. Proportion of girls meeting the MVPA guidelines of 60 min per day
4. Mean minutes of sedentary behaviour (overall and weekday and weekend split)

### Self-reported physical activity and sedentary behaviour

Self-reported physical activity will be captured using the Physical Activity Questionnaire for Adolescents (PAQ-A). The PAQ-A is a 7-day recall used to assess general physical activity levels during the school year using nine items [34]. The mode of commuting to and from school will be assessed using an adapted version of the questionnaire employed in the ENERGY project [35]. Sedentary behaviour will be captured using an adapted version the Adolescent Sedentary Activity Questionnaire (ASAQ) [36]. Participants will report the time they devote to a variety of different sedentary behaviours (e.g., watching TV, using the computer for fun, homework, reading, sitting around with friends etc.) in their free time on a typical weekday and weekend day.

### Demographic and anthropometric measures

Participants will be asked to report their date of birth, ethnicity and postcode. Postcode will be used to calculate the Index of Multiple Deprivation. Standing and sitting height will be measured using a portable stadiometer and weight and body fat with body composition scales (Tanita SC330S). Body mass index (BMI) will be calculated and converted to a BMI percentile based on UK reference data [37]. Stature (i.e., standing height), sitting height, leg length (stature-sitting height), age, and their interactions will be used to predict how many years a girl is from age at peak height velocity (APHV) [38], an indicator of biological maturity. APHV will be used categorise girls into maturity groups (early, average and late maturing).

### Psychosocial measures

Several physical activity-related psychosocial measures will be collected. Enjoyment of physical activity (16 items) [39], motivation (20 items) [40] and confidence to take part in physical activity (8 items) [41], social support for physical activity from family (3 items) and friends (5 items) [42] and attitudes towards being active (14 items) [43] will be assessed. All of these questionnaires include a five point likert scale, apart from social support (four point). Questions on global self-esteem (6 items), physical self-worth (5 items) and body attractiveness (7 items) will be adapted from the physical self-perception profile questionnaire [44]. Participants will also be asked about their intentions to be active for at least 60 min every day during the next month using three items on a 7-point likert scale (from very unlikely to very likely) adapted from Hagger and colleagues [45]. Perceived autonomy support during physical education will be assessed with six items from the adapted version of the Sport Climate Questionnaire [46]. Responses will be reported on a 7-point likert scale from strongly disagree to strongly agree.

### Negative health behaviours

Data on smoking and alcohol will be assessed using questions from the Health Survey for England [47]. Participants will be asked if they have ever tried smoking a cigarette (yes/no) and if they have, how often have they smoked as well as if they have ever drank an alcoholic drink (yes/no) and how often.

### School environment measures

Participant’s perceptions of the school physical activity environment will be assessed using a slightly modified version of the Questionnaire Assessing School Physical Activity Environment (Q-SPACE-R) [48]. The Q-SPACE-R has 16 items, eight assess the school’s physical, physical activity environment (i.e., equipment, facility quality and programming) and eight assess the school’s social physical activity environment. Answers are given on a five point likert scale from strongly agree to strongly disagree.

Lead PE teachers will complete a school characteristics questionnaire containing the following questions: 1) availability, opportunities and access to physical activity and recreation facilities; physical activity, PE and sport policies and practices; and the structure of PE classes and clubs (i.e. male and female mixed or one sex only). These questions were adapted from the ISCOLE school administrator questionnaire [49]; 2) an inventory on the types of physical activities offered by the school. These questions were adapted from the PE and Sport Survey [50]; 3) an inventory of other programmes that are offered by the school to girls in Key Stage 3; and 4) number of teachers, PE teachers and PE support staff in the school. Questions were in the format of multiple choices with free text space to allow the teachers to expand on their answers. These will be followed up with supporting questions in the process evaluation.

### Process evaluation

Process evaluation will be used to help explain any discrepancies between expected and observed outcomes, to understand the influence of context on study outcomes and to elucidate what specific components of Girls Active are deemed a success by pupils and teachers to help inform future intervention development and implementation [51]. Process evaluation will be undertaken throughout the intervention from project initiation to conclusion. We will employ a variety of techniques (e.g., observations, log books, questionnaires, interviews and focus groups with teachers and girls) to record information on recruitment, the implementation/delivery of the intervention, the extent to which the intervention reached the intended targets and the degree to which the targets engaged with ‘Girls Active’ (dose, fidelity, reach and exposure). We will also document any environmental factors that may impact on the study. This may include changes in local or national physical activity and school sport policies, contamination by other similar programmes offered, movement of any teachers from intervention to control schools and secular trends) that may have an influence on intervention effectiveness. As intervention acceptability predicts continued use of intervention strategies [52], student enjoyment and teacher acceptability will also be assessed. Details of the process evaluation components are presented in Table 1.

**Table 1:** Outline of process evaluation

Indicators	Data sources	Timing
**Recruitment**		
Number of schools invited, number of school accepting invitation	Project records, include socio-demographic information (e.g., school size, ethnicity, SES)	Ongoing throughout project
Number of possible participants at each school, number of participants recommended or invited to attend activities, actual number who do attend each activity	School rolls, project records, attendance records	
Number who opt out	Attendance records; survey to explore reasons	
**What was delivered**		
Number of activities delivered, changes to school policy, number of peer leaders recruited, resource use, funding applied for, training conducted and attendance at training	School 'Mission Analysis' self-review and action plan, school environment questionnaire, teacher and peer mentor logs, policy review, project records for funding and training, interviews with lead teacher and peer mentors, logs from and interviews with intervention deliverers, audit of school provision	Monthly collection of logs/records, brief interviews at regular intervals throughout project, final exit interviews including review of initial 'mission analysis', end of intervention policy review and school environment questionnaire (pre-, post-)
**Description of unintended events**		
It is useful to note whether there were any unexpected side effects or outcomes from the intervention. For example, did participants take up one type of physical activity but stop doing another during the project? Unexpected outcomes do not necessarily have to be negative and there may be unanticipated positive health outcomes.	Survey with pupils, attendance logs	monthly collection of attendance, exit survey
**Participant satisfaction, acceptability and enjoyment**		
Satisfaction/dissatisfaction with the programme; likes and dislikes	Lead teacher interview; teacher focus groups; peer leaders focus groups; pupils brief exit survey to all pupils, focus groups with subset. All conducted by person independent of the intervention delivery to encourage honest opinions	Midpoint (brief) and exit
**Understanding supporting networks**		
What local and national infrastructure exists that schools/lead teachers perceive as useful to support physical activity promotion; extent to which formal or informal networks exist and are used	Interview with lead teachers of control and intervention schools	Midpoint and exit
**Sustainability**		
Whether plans have been made to continue with the intervention in some way	Interview with lead teacher	Exit interview

### Statistical analysis

This study will be analysed and reported according to the CONSORT statement for cluster RCTs. Data will be analysed on a complete case basis. The purpose of the primary analysis is to examine whether objectively measured MVPA at 14 months is higher in the intervention group than the control group. This will be examined using a linear multilevel model with MVPA as the outcome variable, levels to indicate the clustering of pupils within schools, a binary indicator for randomisation group as the explanatory variable, and terms for the stratification factors as confounders. Secondary outcomes, including those measured at other time-points, will be analysed using the same strategy with linear multilevel models used for continuous outcomes and logistic multilevel models used for categorical variables. Any changes in MVPA will be explored by extending the multilevel models to investigate whether the measured individual or school level characteristics mediate these changes. Sub-group analyses involving interaction and stratified analyses will be performed for non-active vs. active participants (defined as below or above the median MVPA level at baseline), single vs. mixed sex schools, white vs. BME participants and the degree of social deprivation based on the English indices of deprivation for the school location. These sub-group analyses will use the same models as the main analysis. Missing data will be replaced using multiple imputation methods. As a sensitivity analysis, we will repeat the analysis on both an intention to treat basis and per-protocol basis. To allow intention-to-treat analyses, missing data will be imputed using multiple imputation methods. The per-protocol analysis will only include those who were compliant with the protocol and follow-up visits. No formal interim analyses are planned. The baseline characteristics of those who did and did not drop-out will be compared to determine whether and how they differ. All tests and reported p-values will be two-sided. Estimates will be presented with 95 % confidence intervals.

### Cost-effectiveness analysis

In this economic analysis we will fully cost the delivery of the ‘Girls Active’ intervention and the associated costs such as teacher time and other materials used. We will administer a diary to school leads asking them to complete a record of the additional time, or displaced time, taken to offer the ‘Girls Active’ intervention. We will use Local Education Authority teacher costs, accounting for overheads. From a public sector, multi-agency perspective, taking into account NICE guidance on economic evaluation of public health interventions [53], and based on our experience of evaluating the Wales National Exercise referral programme [54, 55], we will undertake a primary cost-effectiveness analysis of the ‘Girls Active’ intervention, using minutes of MVPA as the outcome effect, and a secondary cost per Quality Adjusted Life Years (QALY) analysis (embedded in a wider cost consequence analysis), using the Child Health Utility 9D (CHU-9D) as our source of utility weights [56]. The CHU-9D is a paediatric generic preference based measure of health related quality of life and consists of nine dimensions (worried, sad, pain, tired, annoyed, school work/homework, sleep, daily routine and ability to join in activities). This questionnaire will be completed at all measurement time points. We will pay particular attention to equity considerations through subgroup analysis (age, ethnicity, socio economic status and BMI at baseline). We will account for clustering in our analysis, producing cost-effectiveness planes and cost effectiveness acceptability curves (CEACs) in order to convey to policy makers the probability that ‘Girls Active’ is cost-effective at different payer thresholds. We will undertake 5,000 boot-strapped replications in order to generate confidence intervals around point estimates. In addition to this traditional approach we will also discuss the costs to this geographical area of “shifting the curve” – the cost of potentially shifting a proportion of girls from inactive to moderately active or moderately active to very active, depending on the findings of the trial. We will also undertake a sensitivity analysis to explore the impact of varying key factors on our results. Data on number of days absent from school, number of visits to the GP, school nurse and school counsellor will also be collected from participants.

## Discussion

The primary aim of this study is to evaluate the impact of an intervention specifically targeting the physical activity levels of adolescent girls. Reviews have indicated that successful physical activity interventions in young people have focused on peer tutoring and peer modelling [8, 11]. The ‘Girls Active’ intervention will incorporate this previously successful element as well as focusing on a wider whole school approach through training for teachers, teachers reviewing current physical activity, PE and sport provision for girls, setting action plans and on-going mentoring from the Youth Sport Trust. Considerable time and resources have been spent on developing ‘Girls Active’; it has undergone a thorough iterative development over 10 years with engagement from all relevant stakeholders.

Strengths of this study include the robust randomised controlled trial design with baseline, short term and longer term follow-up, the quantitative and qualitative process evaluation, the rigorous economic evaluation and the use of an objective measure of physical activity as the primary outcome.

The findings of this study will provide valuable information on whether this type of approach to increasing physical activity in adolescent girls is both effective and cost-effective in the UK.

## References

[1] Janssen I, LeBlanc AG (2010). Systematic review of the health benefits of physical activity and fitness in school-aged children and youth. Int J Behav Nutr Phys Activ.

[2] Biddle S, Asare M (2011). Physical activity and mental health in children and adolescents: a review of reviews. Brit J Sport Med.

[3] UK Chief Medical Officers. Start Active, Stay Active: A report on physical activity for health from the four home countries’ Chief Medical Officers: Department of Health; 2011. www.dh.gov.uk/en/Publicationsandstatistics/Publications/PublicationsPolicyAndGuidance/DH_128209. Accessed 17 Nov 2013.

[4] Public Health England. Health Survey for England 2012, Trend Tables: Health and Social Care Information Centre; 2013. http://www.hscic.gov.uk/catalogue/PUB13219. Accessed 18 Nov 2013.

[5] Gorely T, Sandford R, Duncombe R, Musson H, Edwardson C, Kay T et al. Understanding psycho-social attitudes towards sport and activity. In girls: Women’s Sport & Fitness Foundation; 2011. http://www.lboro.ac.uk/microsites/ssehs/youth-sport/downloads/research-downloads/physical-activity-dl/wsff-final-report.pdf. Accessed 15 June 2014.

[6] Sherar LB, Esliger DW, Baxter-Jones A, Tremblay MS (2007). Age and gender differences in youth physical activity: does physical maturity matter?. Med Sci Sports Exerc.

[7] Bailey R, Wellard I, Dismore H. Girls’ participation in physical activities and sports: Benefits, patterns, influences and ways forward; 2004. http://www.icsspe.org/sites/default/files/Girls.pdf. Accessed 7 June 2014.

[8] Camacho-Miñano MJ, LaVoi NM, Barr-Anderson DJ (2011). Interventions to promote physical activity among young and adolescent girls: a systematic review. Health Edu Res.

[9] Kamath CC, Vickers KS, Ehrlich A, McGovern L, Johnson J, Singhal V (2008). Behavioral Interventions to Prevent Childhood Obesity: A Systematic Review and Metaanalyses of Randomized Trials. J Clin Endocrinol Metab.

[10] van Sluijs EMF, McMinn AM, Griffin SJ (2007). Effectiveness of interventions to promote physical activity in children and adolescents: systematic review of controlled trials. BMJ.

[11] Dobbins M, Husson H, DeCorby K, LaRocca RL. School-based physical activity programs for promoting physical activity and fitness in children and adolescents aged 6 to 18. Cochrane Database Syst Rev. 2013;2:CD007651. doi:10.10.1002/14651858.CD007651.pub2. 10.1002/14651858.CD007651.pub2 PMC7197501 23450577

[12] Edwardson C, Gorely T, Musson H, Duncombe R, Sandford R (2014). Does activity-related social support differ by characteristics of the adolescent. J Phys Activ Health.

[13] Coleman L, Cox L, Roker D (2008). Girls and young women's participation in physical activity: psychological and social influences. Health Edu Res.

[14] Harrell J, McMurray R, Gansky S, Bangdiwala S, Bradley C (1999). A public health vs a risk-based intervention to improve cardiovascular health in elementary school children: The Cardiovascular Health in Children Study. Am J Pub Health.

[15] McKenzie TL, Nader P, Strickmiller P, Yang M, Stone EJ, Perry CL, et al. School physical education: effect of the Child and Adolescent Trial for Cardiovascular Health. Prev Med. 1996;25:423–31. 10.1006/pmed.1996.0074 8818066

[16] Sallis J, McKenzie TL, Alcaraz J, Kolody B (1997). faucette N, Hovell MF. The effects of a 2-year physical education program (SPARK) on physical activity and fitness in elementary school students. Am J Pub. Health.

[17] Simons-Morton B, Parcel G, Baranowski T, Forthofer R, O'Hara NM. Promoting physical activity and a healthful diet among children: results of a school based intervention study. Am J Pub Health. 1991;81:986–91. 10.2105/ajph.81.8.986 PMC1405714 1854016

[18] Muller MJ, Asbeck I, Mast M, Langnase K (2001). Prevention of obesity - more than an intention. Concept and first results of the Kiel Obesity Prevention Study (KOPS). Int J Obes Related Meta Disorders.

[19] Verstraete S, Cardon G, De Clercq DLR, De Bourdeaudhuij IMM (2007). A comprehensive physical activity promotion programme at elementary school: the effects on physical activity, physical fitness and psychosocial correlates of physical activity. Public Health Nutr.

[20] Salmon J, Booth ML, Phongsavan P, Murphy N, Timperio A (2007). Promoting physical activity participation among children and adolescents. Epidem Rev.

[21] Timperio A, Salmon J, Ball K (2004). Evidence-based strategies to promote physical activity among children, adolescents and young adults: review and update. J Sci Med Sport.

[22] Gorely T, Nevill ME, Morris JG, Stensel DJ, Neville A. Effect of a school-based intervention to promote healthy lifestyles in 7–11 year old children. Int J Behav Nutr Phys Activ. 2009;6:5. doi:10.1186/1479-5868-6-5. 10.1186/1479-5868-6-5 PMC2637227 19154622

[23] Sahota P, Rudolf MC, Dixey R, Hill AJ, Barth JH, Cade J (2001). Evaluation of implementation and effect of primary school based intervention to reduce risk factors for obesity. BMJ.

[24] Warren J, Henry C, Lightowler H, Bradshaw S, Perwalz S (2003). Evaluation of a pilot school programme aimed at the prevention of obesity in children. Health Promot Int.

[25] Ridgers N, Stratton G, Fairclough S, Twisk J (2007). Long-term effects of a playground markings and physical structures on children's recess physical activity levels. Prev Med.

[26] Stratton G (2000). Promoting children's physical activity in primary school: An intervention study using playground markings. Ergonomics.

[27] Stratton G, Mullan E (2005). The effect of multicolour playground markings on children's physical activity level during recess. Prev Med.

[28] Fairclough SJ, Stratton G (2005). Improving health-enhancing physical activity in girls’ physical education. Health Edu J.

[29] Fairclough SJ, Stratton G (2006). Effects of a physical education intervention to improve student activity levels. Phys Edu Sport Psych.

[30] Moher DHS, Schukz KF, Montori V, Gotzsche PC, Devereaux PJ (2010). CONSORT 2010 Explanation and Elaboration: updated guidelines for reporting parallel group randomised trials. BMJ.

[31] Jago R, Sebire SJ, Cooper AR, Haase AM, Powell J, Davis L (2012). Bristol girls dance project feasibility trial: outcome and process evaluation results. Int J Behav Nutr Phys Activ.

[32] Bandura A (1986). Social foundations of thought and action: A social cognitive theory.

[33] Schaefer CA, Nigg CR, Hill JO, Brink LA, Browning RC (2014). Establishing and evaluating wrist cutpoints for the GENEActiv accelerometer in youth. Med Sci Sports Exerc.

[34] Kowalski K, Crocker P, Kowalski N (1997). Convergent validity of the Physical Activity Questionnaire for Adolescents. Ped Exerc Sci.

[35] van Stralen MM, Te Velde SJ, Singh AS, De Bourdeaudhuij I, Martens MK, van der Sluis M (2011). EuropeaN Energy balance Research to prevent excessive weight Gain among Youth (ENERGY) project: Design and methodology of the ENERGY cross-sectional survey. BMC Public Health.

[36] Hardy LL, Booth ML, Okely AD (2007). The reliability of the adolescent sedentary activity questionnaire. Prev Med.

[37] Cole TJ, Freeman JV, Preece MA (1995). Body mass index reference curves for the UK, 1990. Arch Dis Childhood.

[38] Mirwald RL, Baxter-Jones A, Bailey DA, Beunen GP (2002). An assessment of maturity from anthropometric measurements. Med Sci Sports Exerc.

[39] Motl RW, Dishman RK, Saunders R, Dowda M, Felton G, Pate RR (2001). Measuring enjoyment of physical activity in adolescent girls. Am J Prev Med.

[40] Goudas M, Biddle S, Fox K (1994). Perceived locus of causality, goal orientations, and perceived competence in school physical education classes. Brit J Educ Psychl.

[41] Motl RW, Dishman RK, Trost SG, Saunders RP, Dowda M, Felton G (2000). Factorial validity and invariance of questionnaires measuring social-cognitive determinants of physical activity among adolescent girls. Prev Med.

[42] Davison KK (2004). Activity-related support from parents, peers, and siblings and adolescents' physical activity: are there gender differences?. J Phys Act Health.

[43] Nelson TD, Benson ER, Jensen CD (2010). Negative attitudes toward physical activity: Measurement and role in predicting physical activity levels among preadolescents. J Ped Psych.

[44] Whitehead J (1995). A study of children’s physical self-perceptions using an adapted physical self-perception profile questionnaire. Ped Exerc Sci.

[45] Hagger M, Chatzisarantis NL, Hein V, Soós I, Karsai I, Lintunen T (2009). Teacher, peer and parent autonomy support in physical education and leisure-time physical activity: A trans-contextual model of motivation in four nations. Psych Health.

[46] Hagger MS, Chatzisarantis NL, Culverhouse T, Biddle SJ (2003). The processes by which perceived autonomy support in physical education promotes leisure-time physical activity intentions and behavior: a trans-contextual model. J Edu Psych.

[47] Health and Social Care Information Centre. Health Survey for England 2011: Methods and Documentation; 2012. http://www.hscic.gov.uk/catalogue/PUB09300/HSE2011-Methods-and-docs.pdf. Accessed 14 April 2014.

[48] Martin JJ, McCaughtry N, Flory S, Murphy A, Wisdom K (2011). Validity and reliability of the school physical activity environment questionnaire. Meas Phys Educ Exerc Sci.

[49] Katzmarzyk PT, Barreira TV, Broyles ST, Champagne CM, Chaput J-P, Fogelholm M (2013). The International Study of Childhood Obesity, Lifestyle and the Environment (ISCOLE): design and methods. BMC Public Health.

[50] Department of Education. PE and School Sport Survey 2009/10; 2010. https://www.gov.uk/government/uploads/system/uploads/attachment_data/file/181556/DFE-RR032.pdf. Accessed 9 Dec 2014.

[51] Craig P, Dieppe P, Macintyre S, Michie S, Nazareth I, Petticrew M (2008). Developing and evaluating complex interventions: the new Medical Research Council guidance. BMJ.

[52] Elder JP, Lytle L, Sallis JF, Young DR, Steckler A, Simons-Morton D, et al. A description of the social–ecological framework used in the trial of activity for adolescent girls (TAAG). 2007;22:155–165. 10.1093/her/cyl059 PMC2764407 16855014

[53] National Institute for Health and Care Excellence (2012). Methods for the Development of NICE Public Health Guidance.

[54] Edwards RT, Linck P, Hounsome N, Raisanen L, Williams N, Moore L (2013). Cost-effectiveness of a national exercise referral programme for primary care patients in Wales: Results of a randomised controlled trial. BMC Public Health.

[55] Murphy SM, Edwards RT, Williams N, Raisanen L, Moore G, Linck P (2012). An evaluation of the effectiveness and cost effectiveness of the National Exercise Referral Scheme in Wales, UK: a randomised controlled trial of a public health policy initiative. J Epidemiol Comm Health.

[56] Stevens KJ (2011). Assessing the performance of a new generic measure of health related quality of life for children and refining it for use in health state valuation. Am J Pub Health.

